# A mutation operator self-adaptive differential evolution particle swarm optimization algorithm for USV navigation

**DOI:** 10.3389/fnbot.2022.1076455

**Published:** 2022-12-06

**Authors:** Yuehong Gong, Shaojun Zhang, Min Luo, Sainan Ma

**Affiliations:** ^1^School of Navigation and Shipping, Shandong Jiaotong University, Weihai, China; ^2^School of Information Science and Engineering, Harbin Institute of Technology at Weihai, Weihai, China; ^3^Zhejiang Jialan Ocean Electronics Co., Ltd., Zhoushan, China

**Keywords:** differential evolution algorithm, hybridization, mutation, particle swarm optimization, unmanned surface vessel path planning, scaling factor

## Abstract

To keep the global search capability and robustness for unmanned surface vessel (USV) path planning, an improved differential evolution particle swarm optimization algorithm (DePSO) is proposed in this paper. In the optimization process, approach to optimal value in particle swarm optimization algorithm (PSO) and mutation, hybridization, selection operation in differential evolution algorithm (DE) are combined, and the mutation factor is self-adjusted. First, the particle population is initialized and the optimization objective is determined, the individual and global optimal values are updated. Then differential variation is conducted to produces new variables and cross over with the current individual, the scaling factor is adjusted adaptively with the number of iterations in the mutation process, particle population is updated according to the hybridization results. Finally, the convergence of the algorithm is determined according to the decision standard. Numerical simulation results show that, compared with conventional PSO and DE, the proposed algorithm can effectively reduce the path intersection points, and thus greatly shorten the overall path length.

## 1. Introduction

With the increasing demands of marine monitoring and exploration, the unmanned surface vessel (USV) becomes widely used in water area environmental inspection. The main research direction in the application of the USV is flight path planning. The purpose of path planning is to obtain a scientific, safe, and concise path in a specific environment. As the USV navigation environment deteriorates, the problems needed to be solved in USV path planning are becoming harsher, and the amount of calculation is also increasing, so the path planning problem gets trickier. In recent years, many studies has have been done for USV flight path planning and swarm intelligence algorithm has been popularly used, including particle swarm optimization (PSO) (Xin et al., 2019; Krell et al., 2022), artificial fish swarm algorithm (Zhao et al., 2022), ant colony algorithm (Wang et al., 2022), genetic algorithm (GA) (Park et al., 2021), and etc. Among these intelligent algorithms, particle swarm optimization is the most widely used in the field of automatic control because of its simple principle, fewer parameters, fast optimization speed, small amount of calculation and other advantages (Khayati et al., 2019). At present, the application of particle swarm optimization algorithm in robot path planning is very active (Chen and Sun, 2021; Wu et al., 2022; Xiao et al., 2022), but the research on USV flight path planning is still a frontier field. In practical application, due to the irregular, complex and uncertain USV navigation environment, the algorithm is easy to fall into local extreme values, resulting in poor quality of generated paths, waste of time and resources. Therefore, improving the global search ability and robustness of the algorithm becomes a key content in USV flight path planning. The most popular use of PSO for global path planning is genetic particle swarm optimization (GaPSO), which combines the advantages of the genetic algorithm (GA) and the particle swarm optimization algorithm. In Pehlivanoglu (2012), an improved PSO for robot path planning is presented. In Zhang and Xing (2019), GA is combined with the voronoi diagram to generate the optimal path for autonomous unmanned aerial vehicles (UAVs). The applying of GaPSO can reduce the probability of falling into local optimal solutions, however, the global search capability needs to be enhanced. Differential evolution algorithm (DE) has been used in many fields such as intelligent machines, robots, equipments and so on Yang et al. (2011) and Yildiz (2013). Because of its strong global searching ability and high robustness, DE can be used to solve multiobjective, constrained and complex optimization problems.

Compared with genetic algorithms, the global exploration ability of DE is more obvious. However, the accuracy of the optimal solution and convergence speed are affected by the parameter settings and the mutation operations. To get a compromise between the optimal solution accuracy and optimization speed, many studies have been done on the values of the control parameters, including the population size *NP*, the crossover probability *CR*, and the scaling factor *F*. It is well known that the size of the population increases, the probability of finding the optimal solution is higher, however the amount of calculation and computation time may also increase. The value of *F* has a great influence on the speed of optimization. For a larger *F* value, the population is more abundant, but the amount of calculation is larger and the computation time is longer. With a smaller *F*, the optimal solution can be found faster, but the diversity of the population can't be guaranteed, so the algorithm will easily fall into a local optimum value. To obtain appropriate parameters, a lot of studies have been done. In Yildiz (2013), the authors pointed out that *F* should be set to 0.5. In Li et al. (2019), the authors suggested that *F* should between [0.4, 0.95]. The values of these parameters are difficult to select to obtain a better performance.

In order to get a better flight path with fast optimization speed and less computation, this paper proposed a discrete particle swarm optimization algorithm with differential evolution algorithm (DeDSO) for USV flight path planning. This algorithm combines the advantages of the conventional particle swarm optimization algorithm and the differential evolution algorithm, so as to enhance the global search ability and improve the path planning efficiency and stability. In order to ensure both high search speed and high precision solution, the scaling factor *F* in DE is self-adaptive with the iteration.

The remainder of this paper is organized as follows. Section 2 gives the mathematical model of USV automatic inspection problem. Section 3 introduces the working principle of conventional PSO, DE, and the improved DePSO. Section 4 gives the framework and implementation procedure of the DePSO. Section 5 describes the numerical simulation results and comparison. Finally, section 6 gives a short conclusion.

## 2. Mathematical model of USV automatic inspection problem

**Figure 1:**
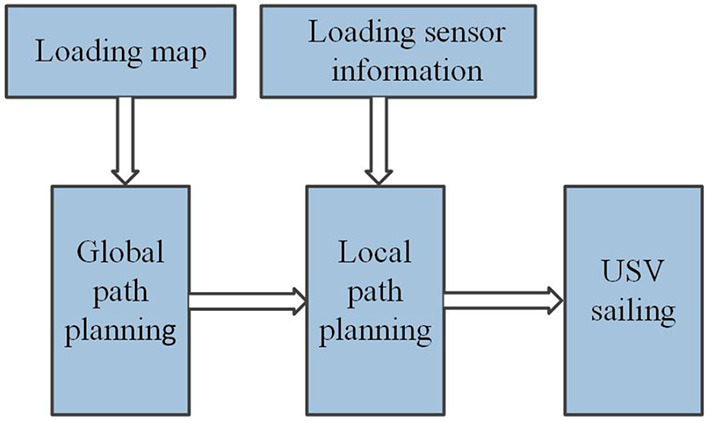
USV path planning process diagram.

Flight path planning is a global optimization problem aims to search the optimal flight path for USVs. When USV is applied in water environment inspection, it is necessary to generate a sailing path. The path planning process of the USV is shown in Figure 1. Firstly, a feasible global path is drawn according to the regional map information by identifying the surrounding environment and the status information of the USVs. Then the ship navigates according to the specified path. During the navigation, the sensors monitor the surrounding environment, and the local path planning is used to avoid obstacles, and then the ship continues to travel according to the original path.

The problem to be solved in this paper is to plan a global path for USVs applied in water area automatic inspection. In a certain water area, several sampling points are spread, which will be automatically inspected and sampled by the USVs. The purpose of flight path planning is to generate a route between the starting point and the destination, which ensures that all sampling points are inspected only once except the starting point, and the path formed should meet specific optimization objectives, which is equivalent to the traveling salesman problem (TSP) (Laporte, 2010; Zheng et al., 2022). The TSP model has been mainly used for single vehicle operating situations as described in this paper, and the main idea is to search the optimal path to visit all sampling points.


(1) $$\begin{array}{l} \min f=\overset{n-1}{\underset{i=1}{\sum }}D\left(i,i+1\right)+D\left(1,n\right) \end{array}$$

The objective function is the key point of an optimization problem. In our design, the optimization objective is demonstrated as function *f*, and the mathematical model corresponding to *n* sampling points is defined as Equation (1). Where D(*i*, *i* + 1) respects the distance between sampling point “*i*” and sampling point “*i* + 1.” In this paper, the USV sampling problem is corresponding to symmetry TSP problem, which satisfies D(*i*, *j*) = D(*j*, *i*), where *i*, *j* ∈ (1, 2, …, *n*).

## 3. Improved particle swarm optimization model

### 3.1. Particle swarm optimization algorithm

**Figure 2:**
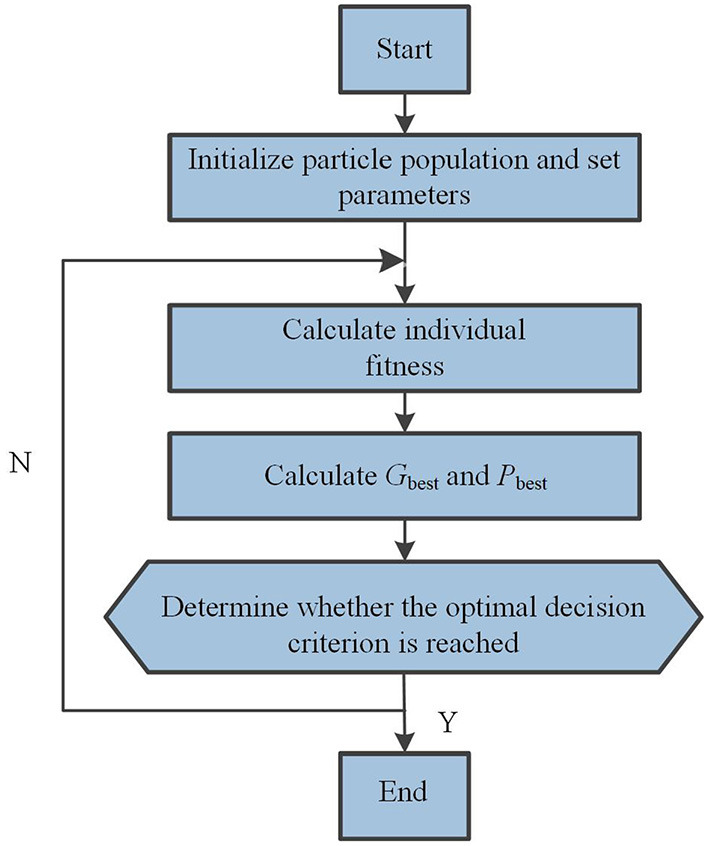
The optimization process of PSO.

In particle swarm optimization, the updating of population tends to be closer to the optimal value. The optimization process of PSO is shown in Figure 2. Particles adjust their speed and direction of motion according to their own historical optimal value *P*_best_ and global optimal value *G*_best_, and measure the merits of particles by optimizing the fitness value determined by the target, so as to drive the particles to the optimal value (Wu et al., 2017; Rauf et al., 2020).

The next generation particle position *X*_id_(*t*+1) and velocity *V*_id_(*t*) are calculated through Equations (2) and (3) respectively. In which, *X*_id_(*t*) is the *t*th generation particle position; ω is the inertia weight, which determines the optimization speed of the algorithm; *c*_1_ and *c*_2_ decide the speed for the particle approaching *P*_best_ and *G*_pest_ respectively; *R*_1_ and *R*_2_ are random numbers, and their values range from 0 to 1; In this paper, the fitness represents the length of the USV navigation route, the calculation formula is (4), in which (*x*_i_, *y*_i_) are the coordinates of the *i* th and the *i*+1 th sampling point, respectively. The pseudo code of PSO is presented in Table 1.

**Table 1:** Algorithm I: Conventional PSO.

**Conventional particle swarm optimization**
1. Set fitness function *f*, set population size *N*, acceleration coefficients
*c*_1_, *c*_2_, *R*_1_, *R*_2_, inertia weight ω, and maximum iteration number *T*_max_
2. Generate the initial population
3. **For** *t* from 1 to *T*_max_ **do**
4. **For** *i* from 1 to N **do**
5. Initialize velocity and position
6. Evaluate initial fitness value
7. Record initial *P*_pest_ and *G*_pest_
8. **end**
9. Refresh velocity and position from Equations (1) and (2)
10. Refresh the fitness of each particle
11. Update *P*_pest_ and *G*_pest_
12. **If** *t* =*T*_max_ or minimum error criteria is achieved **do**
13. Output the particle with best fitness value
14. **end if**
15. **end**


(2) $$\begin{array}{l} X_{id}\left(t+1\right)=X_{id}\left(t\right)+V_{id}\left(t+1\right) \end{array}$$

$$\begin{array}{l} v_{id}\left(t+1\right)=\omega v_{id}\left(t\right)+c_{1}R_{1}\left(P_{best}-X_{id}\right)\; \end{array}$$

(3) $$\begin{array}{l} +c_{2}R_{2}\left(G_{best}-X_{id}\right) \end{array}$$

(4) $$\begin{array}{l} f\left(x\right)=\overset{n-1}{\underset{i=1}{\sum }}\sqrt{{\left(y_{i+1}-y_{i}\right)}^{2}+{\left(x_{i+1}-x_{i}\right)}^{2}} \end{array}$$

### 3.2. Differential evolution algorithm

**Figure 3:**
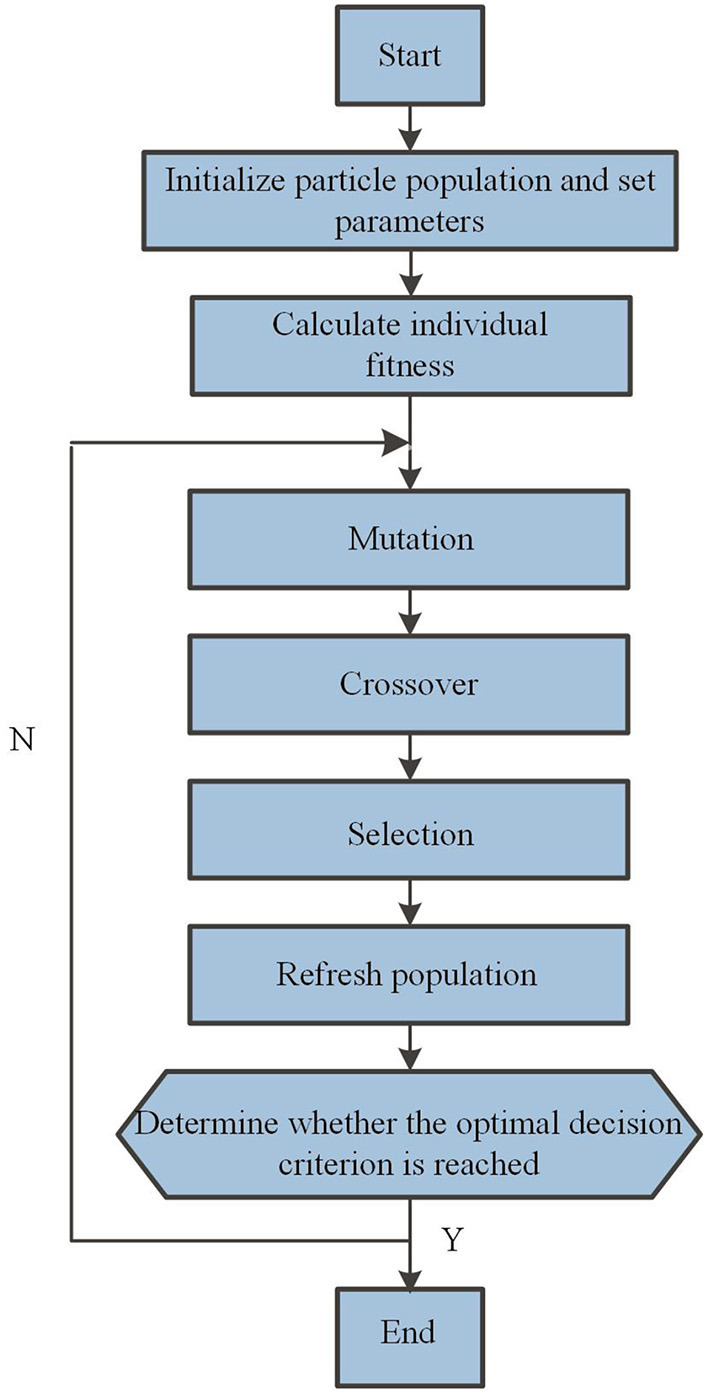
The optimization process of DE.

Differential evolution algorithm is a bionic intelligent calculation method which simulates the natural evolutionary law to find the global optimal value. The optimization process of DE is shown in Figure 3. The main body of the algorithm generally includes three steps: mutation, crossover and selection (Yi et al., 2016; Bilal et al., 2020). The pseudo code of DE is presented in Table 2. Three different individual vectors are randomly selected to get a difference vector, denoted as *X*_r1_, *X*_r2_, and *X*_r3_, and the mutation vector *V*_i_ was calculated according to Equation (5). The scaling factor *F* is set to control the influence of difference vector (*X*_r2_-*X*_r3_) on the evolution speed, and the value of *F* is generally between [0,1].

**Table 2:** Algorithm II: Conventional DE.

**Conventional differential evolution algorithm**
1. Set fitness function, population size *PN*, scaling factor *F*, and cross-
over probability *CR*
2. Generate the initial population
3. **For** *t* from 1 to *T*_max_ **do**
4. **For** *i* from 1 to *NP* **do**
5. Mutation: select *X*_r1_, *X*_r2_ and *X*_r3_ randomly
6. Create the mutation vector *V*_i_ from Equation (5)
7. Crossover: the crossover operation is executed between a
parent individual and mutation vector from Equation (6)
8. Selection from Equation (7)
9. Refresh fitness value
10. **end**
11. **If** *t* =*T*_max_ or minimum error criteria is achieved **do**
12. Output the particle with best fitness value
13. **end if**
14. **end**


(5) $$\begin{array}{l} V_{i}=X_{r1}+F\left(X_{r2}-X_{r3}\right) \end{array}$$

The function of crossover operation is to hybridize the current individuals of the population with the mutation vector generated by the mutation, and generate new individuals randomly according to a certain probability, as shown in Equation (6). *CR* is the crossover probability, which determines the size of the hybridization probability, and its value is a real constant between [0,1].


(6) $$u_{ij}(t+1)=\left\{ \begin{array}{l} V_{ij}(t+1),\;if\;rand\text{(}0,1)\leq CR,\\ \text{or j}=\text{j and}\\ X_{ij}(t),\;else \end{array}\right.$$

The selection operation is based on the individual optimization objective to select one of the next generation individuals between the new vector generated by the crossover and the current vector. If the individual optimization objective of the new vector is better than the current vector, the new vector will be retained. Otherwise, the current vector individual will be inherited to the next generation. The selection formula is shown in Equation (7). In which, *X*_i_(*t*) is the particle position before the *t* th iteration, *U*_i_(*t*) is the new particle position after the differential evolution mutation operation of the *t* th iteration. *X*_i_(*t* + 1) is the particle position after the *t* th iteration. *f*(*X*_i_(*t*)) is the fitness value for particle position *X*_i_(*t*), *f*(*U*_i_(*t*)) is the fitness value for particle position *U*_i_(*t*).


(7) $$X_{i}(t+1)=\left\{ \begin{array}{l} u_{i}(t),if\;f\text{(}u_{i}\text{(t))}<f\text{(}X_{\text{i}}(t)\text{)}\\ X_{i}(t),\;if\;f\text{(}u_{i}\text{(t))}\geq f\text{(}X_{\text{i}}(t)\text{)} \end{array}\right.$$

### 3.3. Differential evolution particle swarm optimization

In the process of population updating, the DePSO iterates from the formula of the particle swarm optimization algorithm to get a new generation of individuals, and then hybridizes them from the formula of the differential evolution algorithm. The object of hybridization is the individual optimal value *P*_best_ and the global optimal value *G*_best_, the selection formula after crossing is shown in Equations (8) and (9).

In order to find a better balance between optimal solution accuracy and convergence speed, the zoom factor *F* is adjusted adaptively with the number of iterations. The improved formula is demonstrated in Equation (10), in which *F*_max_ and *F*_min_ are the maximum and minimum values of the zoom factor, *T*_max_ is the maximum number of iterations, and *t* is the current number of iterations.


(8) $$X_{i}(t+1)=\left\{ \begin{array}{l} u_{i}(t),if\;f\text{(}u_{i}\text{(t))}<f\text{(}P_{\text{best}}\text{)}\\ X_{i}(t),\;if\;f\text{(}u_{i}\text{(t))}\geq f\text{(}P_{\text{best}}\text{)} \end{array}\right.$$

(9) $$X_{i}(t+1)=\left\{ \begin{array}{l} u_{i}(t),if\;f\text{(}u_{i}\text{(t))}<f\text{(}G_{\text{best}}\text{)}\\ X_{i}(t),\;if\;f\text{(}u_{i}\text{(t))}\geq f\text{(}G_{\text{best}}\text{)} \end{array}\right.$$

(10) $$F=\frac{\left(F_{\max}-F_{\min}\right)}{2}(1+\frac{T_{\max}-t}{T_{\max}})$$

## 4. USV path planning problem

### 4.1. PSO applying in TSP problems

In the TSP issue corresponding to automatic inspection and sampling of unmanned pollution detection vessel, a huge number of paths will be generated during flight path planning, and the number of paths will increase factorially with the number of sampling points. A path is equivalent to a particle in PSO, the position of the particle is represented by the path sequence composed of the distribution of sampling points, and the velocity of the particle is represented by the commutator. The calculation process of optimization is realized by exchanging the position of sampling points.

**Figure 4:**
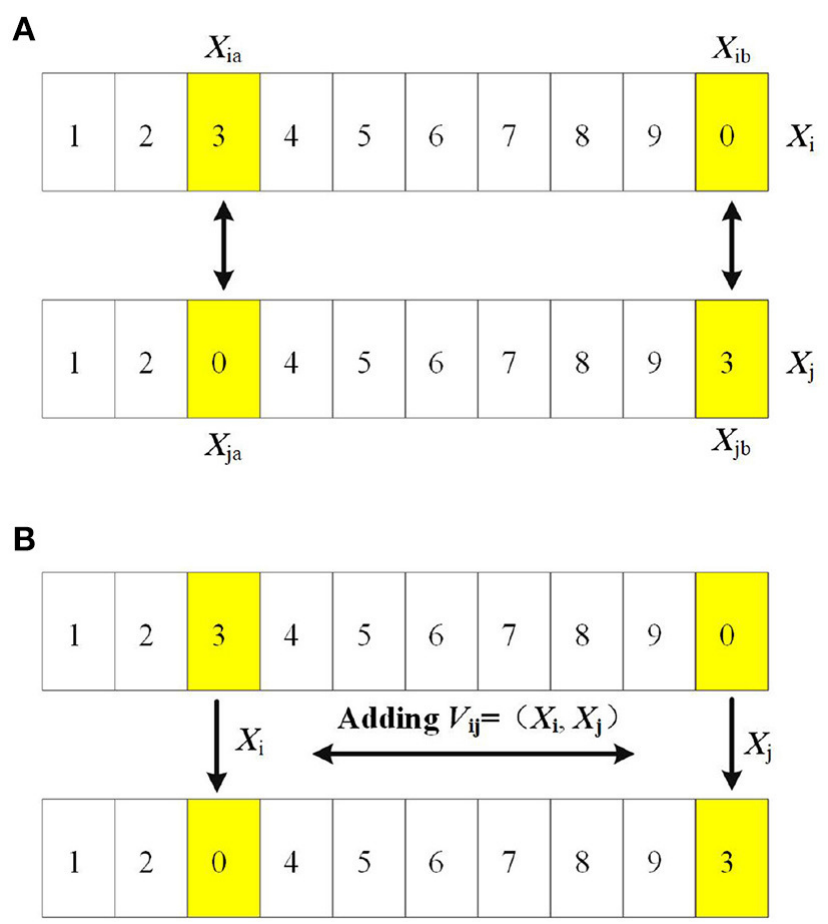
**(A)** Exchange operator. **(B)** Addition of position and velocity.

**Exchange operator**: for two path sequences *X*_i_ = (*X*_i1_, *X*_i2_, …, *X*_n_) and *X*_j_ = (*X*_j1_, *X*_j2_, …, *X*_jn_), if the two sequences have different value at the same position, that is *X*_ia_ ≠ *X*_ja_, thus *V*_ij_ = (*X*_ia_, *X*_ja_) is called the exchange operator for the path sequence, as shown in Figure 4A.

**Exchange sequence**: a sequence composed by the exchange operators, such as *V*= (*V*_1_,*V*_2_…,*V*_n_), in which *n* is the number of locations corresponding to two cities with the same sequence but different values.

**Position of the particle**: the position of the particle is determined by the sequence of cities *X*= (*X*_1_, *X*_2_…, *X*_m_), in which *m* is the number of the cities.

**Velocity of the particle**: the velocity of the particles is given by the exchange sequence *V*= (*V*_1a_, *V*_2b_…, *V*_mn_), in which *V*_mn_ is the exchange operator.

**Addition of position and velocity**: in TSP, we form a new path sequence by the addition of position and velocity to the parent individual, as shown in Equation (11). On the basis of the original path sequence, the new path sequence is obtained by exchanging the city position according to the exchange operator, as shown in Figure 4B.


$$\begin{array}{l} X=\left(X_{1},X_{2},\cdot \cdot \cdot ,X_{\text{i}},\cdot \cdot \cdot X_{\text{j}},...,X_{\text{n}}\right)+{V_{\text{i}}}_{\text{j}}\left(X_{\text{i}},X_{\text{j}}\right)\; \end{array}$$

(11) $$\begin{array}{l} =\left(X_{1},X_{2},\cdot \cdot \cdot ,X_{\text{j}},\cdot \cdot \cdot ,X_{\text{i}},\cdot \cdot \cdot ,X_{\text{n}}\right) \end{array}$$

**Subtraction between positions**: the subtraction from a position to another position forms an exchange sequence that generates a new velocity. To form *V*_ij_ = *X*_i_ − *X*_j_, where *X*_i_ and *X*_j_ are the number of the cities. First we find the city in the 2th sequence which is the same to the 1st element in the 1st sequence, and form an exchange operator *V*(1,i). Then conduct this exchange operator to the first sequence and get a new 1st sequence. Later, find the first position in the 2st sequence with the same element in the new 1st sequence, and get an exchange operator *V*(2,i), this course will continue sequentially until the exchange sequence of two city sequences is obtained, as is shown in Equation (12).


(12) $$\begin{array}{l} \;X_{1}\left(4,3,6,2,1,5,7,8,9,0\right)-X_{2}\left(3,4,6,2,1,5,7,8,9,0\right)\\ =V\left(v\left(1,5\right)+v\left(2,6\right)+v\left(3,6\right)+v\left(4,6\right)+v\left(5,6\right)\right) \end{array}$$

**Scalar multiplication of velocity**: the scalar multiplication of velocity has a probabilistic meaning. For example, when *V*_im_=c*V*_ja_, the selection formula is shown in (13), in which *c* is a constant. During the calculation course, a random number rand is generated for each operator of *V*_ja_, compare *c* and rand, the value of *V*_im_ is determined by the comparison result.


(13) $$V_{\text{im}}=\left\{ \begin{array}{l} V_{\text{ja}},if\text{ r and}<\text{c}\\ \text{0, else} \end{array}\right.$$

### 4.2. DePSO applied in USV path planning

**Figure 5:**
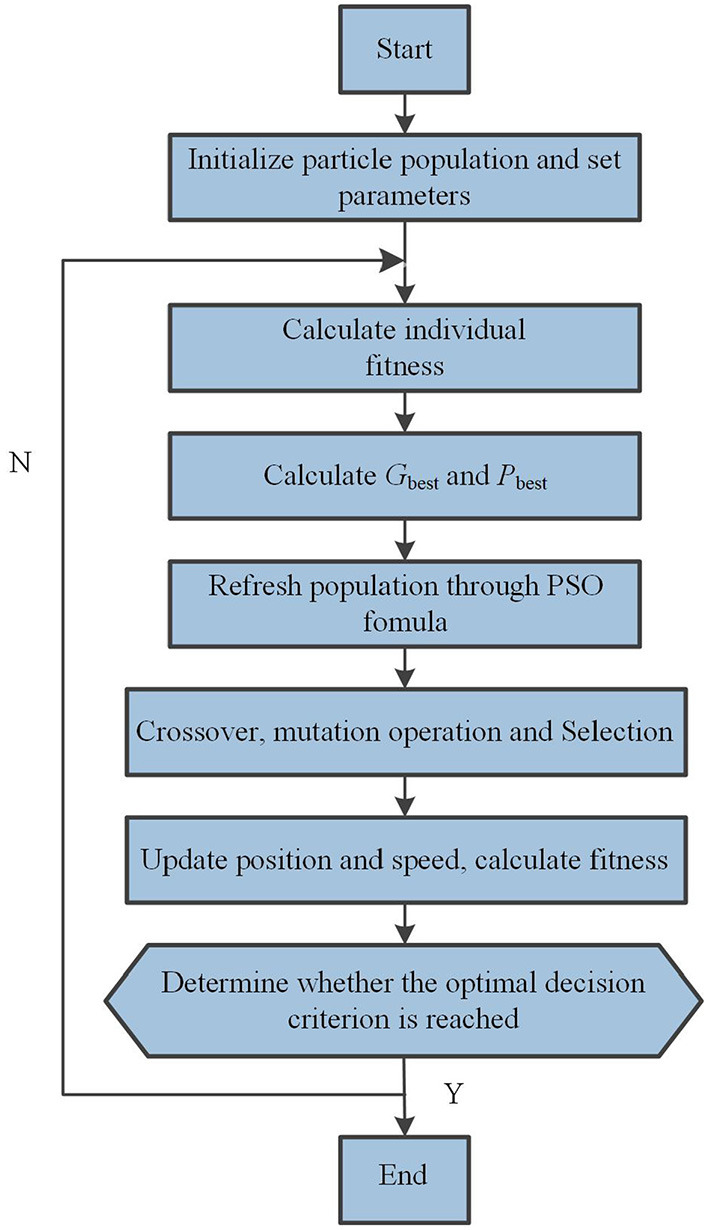
The optimization process of DePSO.

The improved differential evolution particle swarm optimization algorithm is applied to the automatic inspection sampling path planning process of USV. The initial particles are chosen randomly from the possible paths generated at the beginning, and the optimal path is found by the approach of optimal value of DePSO. In the updating and iteration process of each generation of particle population, the idea PSO and DE are combined. The specific implementation steps of the algorithm are shown in Figure 5, and the pseudo code is presented in Table 3.

Step 1: Modeling the environment according to the map model and the distribution of sampling points.
Step 2: Randomly select a certain number of path sequences as the initial particle population, and set the parameter values used in the algorithm.
Step 3: Refresh the population and calculate the fitness value of the particles;
Step 4: Find the individual optimal path *P*_best_ and the population optimal path *G*_best_;
Step 5: Generate mutation vector according to mutation formula of differential evolution algorithm, and cross with *P*_best_ and *G*_best_, respectively to get new path sequence. The fitness of the newly generated path sequence is calculated and compared with the fitness of *P*_best_ and *G*_best_, respectively. If the path becomes shorter, the new particle position is retained, otherwise it is discarded. Update *P*_best_ and *G*_best_ while retaining the position of the new particle.
Step 6: Judge whether the optimal decision is reached; There are two judgment conditions: First, the minimum error criteria of fitness reaches the set accuracy; Second, reach the maximum number of iterations. If the one of the judgment conditions is met, the optimization ends. Otherwise, go back to the second step and the iteration continues.

**Table 3:** Algorithm III: DePSO.

**Improved particle swarm optimization**
1. Set fitness function, population size *NP*, acceleration coefficients
*c*_1_, *c*_2_ and inertia weight ω. Set scaling factor *F*_max_, *F*_min_, and
crossover probability *CR*
2. Generate the initial population
3. **For** *t* from 1 to *T*_max_ **do**
4. **For** *i* from 1 to *NP* **do**
5. Evaluate initial fitness value
6. **end**
7. Record initial *P*_best_ and *G*_best_
8. **For** *i* from 1 to *NP* **do**
9. Refresh the velocity and position from Equations (1) and (2)
10. Refresh fitness value
11. Update *P*_best_ and *G*_best_
12. **end**
13. **For** *P*_best_ and *G*_best_ **do**
14. Mutation: select *X*_r1_, *X*_r2_ and *X*_r3_ randomly
15. Create the mutation vector *V*_i_ from Equation (5)
16. Crossover: the crossover operation is executed between
*P*_best_ or *G*_best_ and mutation vector from Eq. 6
17. Selection from Equations (8) or (9)
18. Refresh fitness value
19. **end**
20. **If** *t* =*T*_max_ or minimum error criteria is achieved **do**
21. Output the particle with best fitness value
22. **end if**
23. **end**

## 5. Numerical simulation results and analysis

In order to verify the flight path planning effect of the improved DePSO for USV, numerically simulation is done to imitation the path planning process. The hardware platform and software environment applied in this experiment is demonstrated in Table 4.

**Table 4:** Experimental hardware and software environment.

**Project**	**Environmental set**
CPU	Intelcore (TM) i7-10510U
Graphics Card	Intel UHD Graphics
Memory	16G
Operational System	Windows 10
Software Version	Matlab 2016R

**Figure 6:**
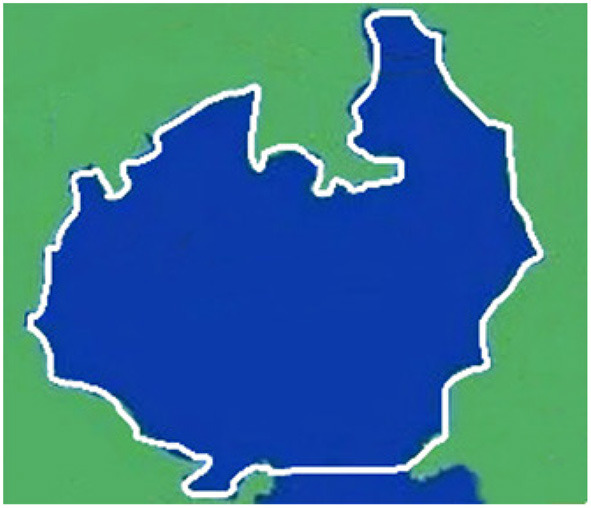
The sea environment map.

**Figure 7:**
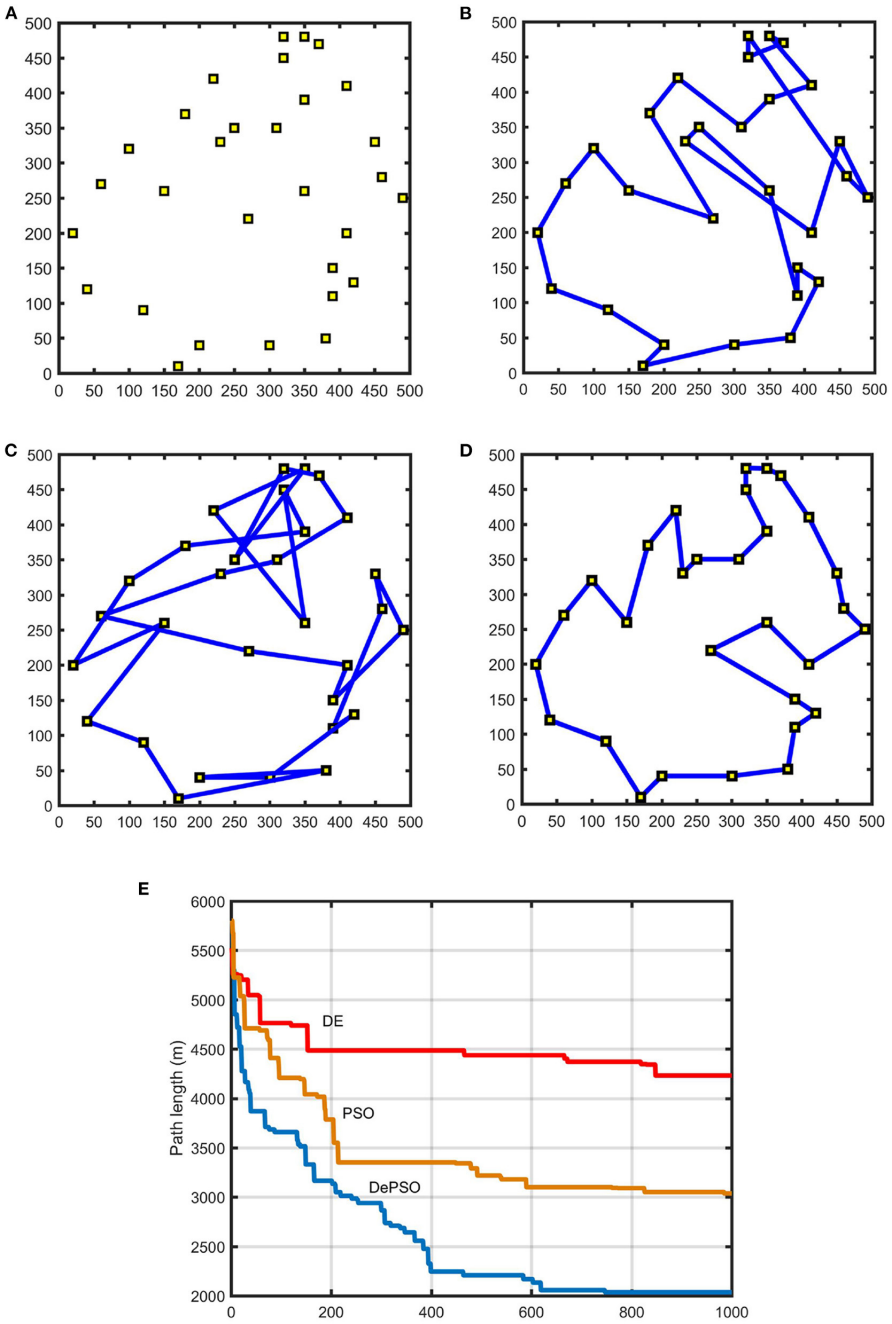
Path planning results for 30 sampling points: **(A)** Distribution of 30 sampling points. **(B)** Path planning result with conventional PSO. **(C)** Path planning result with conventional DE. **(D)** Path planning result with DePSO. **(E)** Path length iterative process comparison.

In this paper, a 500 × 500 m sea environment shown in Figure 6 is chosen as the inspection map model. The simulation environment is set to be ideal. Assuming there are no obstacles in the sea area. The USV is set as a particle without considering the factors such as its volume and position. The environmental factors such as wind and waves are also not considered. Firstly, the USV navigation environment is modeled according to the water environment map and the sampling points distribution. Secondly the improved particle swarm optimization algorithm is applied for global path planning. For comparison, the path planning process of PSO, DE and DePSO were simulated, respectively. The coordinate settings of the 30 sampling points is shown in Figure 7A. The particle population size was selected as 200. The parameter *c*_1_ and *c*_2_ are set to 1, and the inertia weight ω is set to 1. For conventional DE, CR and the zoom factor *F* are set to 1. For DePSO, the maximum value of zoom factor *F*_max_ is set to 1 and the minimum value *F*_min_ is set to 0. Figures 7B–D give the comparison of the final results of route planning with conventional PSO, conventional DE, and DePSO, respectively, under the same map environment with 30 sampling points. Figure 7E gives the path length iterative process comparison for the three methods.

**Figure 8:**
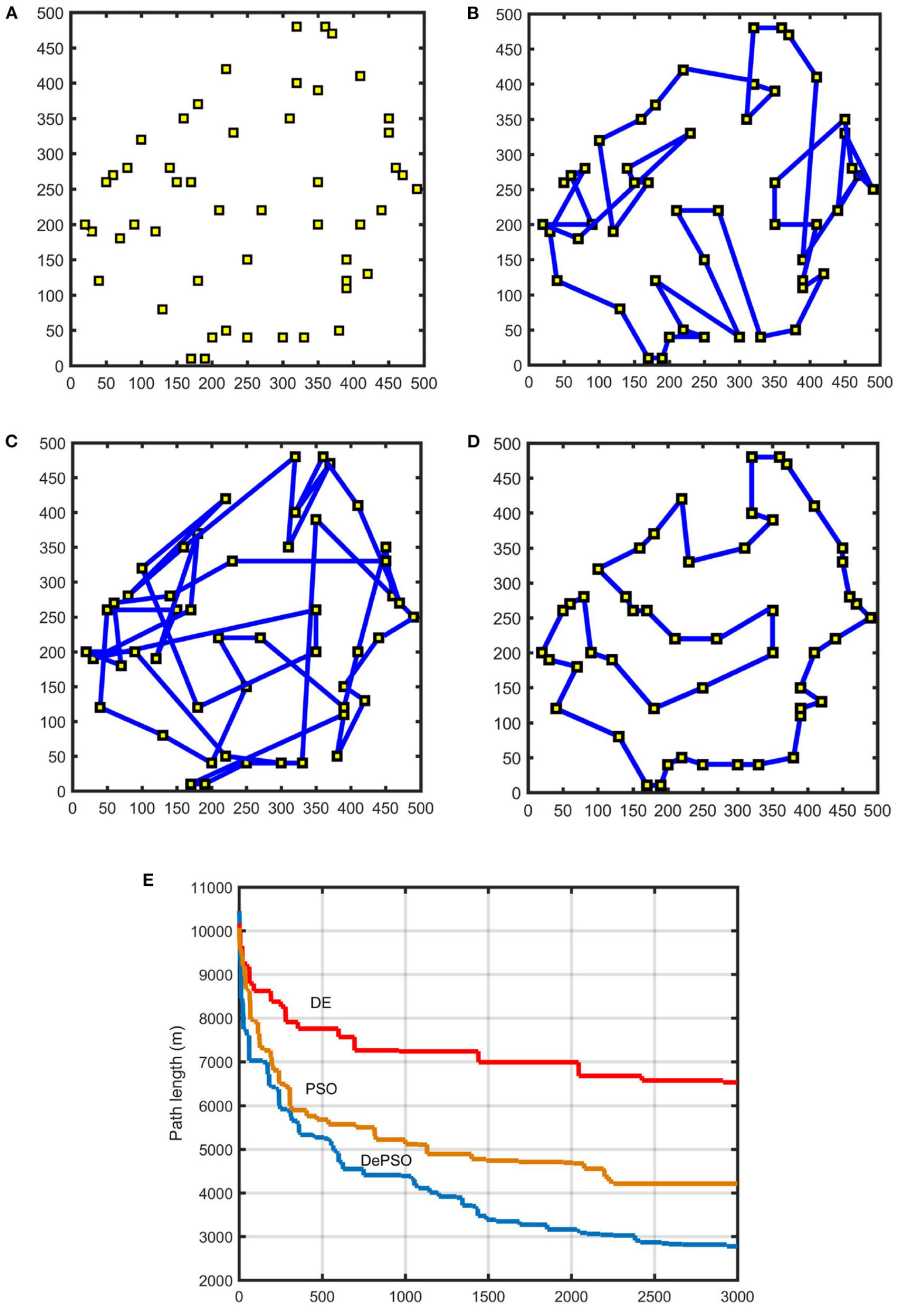
Path planning results for 50 sampling points: **(A)** Distribution of 50 sampling points. **(B)** Path planning result with conventional PSO. **(C)** Path planning result with conventional DE. **(D)** Path planning result with DePSO. **(E)** Path length iterative process comparison.

The coordinate settings of the 50 sampling points is shown in Figure 8A. Figures 8B–D demonstrate the comparison of the path planning results applying conventional PSO, conventional DE, and DePSO, respectively, under the same map environment with 50 sampling points, and Figure 8E gives the path length iterative process comparison.

As the number of sampling points increases, the number of iterations needed to find the optimal path will also increase gradually. In Figure 7, the maximum number of iterations is 1,000. In Figure 8, the maximum number of iterations is 3,000.

As can be seen from the figures, compared with the conventional PSO and DE, by applying the proposed DePSO, the number of overlaps of the final paths is significantly reduced and the total path length is significantly shortened. Because of the combination of the two algorithms, the computation amount for an iteration is increased, so that the computation time is slightly increased. But this increasing time is negligible when comparing with speed enhancement brought by the algorithm improvement.

Table 5 lists the path planning results applying conventional PSO, conventional DE, and DePSO proposed in this paper when the sampling points are 20, 30, 40, 50, and 60, respectively. For each algorithm, 5 times of simulation are done, among which the best, average and worst path length are listed. Applying the conventional PSO and DE algorithms, there are many crossings in the path and the total length of the path lengths are long. Applying the DePSO proposed in this paper, the path complexity is greatly reduced and the path lengths are significantly shorter.

**Table 5:** Comparison of path lengths with PSO, DE, and DePSO.

**Number of sample points**	**Conventional PSO**			**Conventional DE**			**DePSO**		
	**Best**	**Average**	**Worst**	**Best**	**Average**	**Worst**	**Best**	**Average**	**Worst**
20	2,021	2126.4	2,215	2,413	2531.8	2,628	1,874	1911.6	1,939
30	2,769	2879.4	2,978	3,877	3,949	4,023	2,038	2096.8	2,207
40	3,670	3877.8	4,000	5,621	5736.8	5,837	2,391	2401.2	2,408
50	4,020	4102.6	4,195	6,705	6783.8	6,823	2,548	2655.2	2,760
60	4,761	4659.6	4,575	5,464	5,607	5,696	3,022	3086.2	3,186

Table 6 lists the path planning results applying the proposed DePSO with different population size when the sampling point number is 50. For each population size of 100, 200, 300, 400, and 500, 10 times of simulation are done, and the best, average and worst calculation time and path lengths are listed in Table 6. From this table we can get that, when population size rises, the optimization time becomes longer and the path lengths becomes shorter.

**Table 6:** Path planning results with different population sizes.

**Population size**	**Path length (m)**			**Computation time (s)**		
	**Best**	**Average**	**Worst**	**Best**	**Average**	**Worst**
100	2,656	2866.6	3,167	56.88	58.95	59.95
200	2,525	2734.6	2,954	101.09	103.25	112.31
300	2534	2713.0	2903	146.10	150.86	155.32
400	2,529	2642.9	2,876	176.87	181.48	202.03
500	2,453	2629.4	2,742	221.68	243.81	259.94

Table 7 lists the path planning results applying the proposed DePSO with different *F* ranges, when the population size is 200 and the sampling point number is 30. For each *F* range of [0,1], [0.05,0.95], [0.1,0.9], [0.15,0.85], and [0.2,0.8], 10 times of simulation are done, the best, average and worst calculation time and path lengths are listed. From Table 7 we can get that, the optimization result is also affected by the value range of zoom factor *F*. With small *F* range, the optimization course is fast, but the route length is longer. With the larger *F* value, the optimization time becomes longer and the path length is shorter.

**Table 7:** Path planning results with different *F* range.

***F* range**	**Path length (m)**			**Computation time (s)**		
	**Best**	**Average**	**Worst**	**Best**	**Average**	**Worst**
[0,1]	2,038	2074.8	2,150	88.71	89.63	93.88
[0.05,0.95]	2,038	2235.1	2,283	86.75	87.89	90.69
[0.1,0.9]	2,550	2695.2	2,884	83.34	84.08	87.78
[0.15,0.85]	2,893	3,178.8	3,328	83.07	83.43	83.99
[0.2,0.8]	3,537	3772.4	3,905	79.85	81.92	83.94

## 6. Conclusion

Particle swarm optimization algorithm is an effective optimization method for USV global path planning. However, conventional PSO approaches cannot always find the global optima, particularly for complex scenes. DE algorithm has the strong global search ability and good robustness. The combination of PSO and DE can enhance their advantages thus enhance the global search ability of the algorithm. In this paper, an improved particle swarm optimization algorithm (DePSO) was used for global flight path planning of automatic inspection path of USVs. In the optimization process of DePSO, the current vector is differentially crossed with the local optimal value and the global optimal value, which can further enrich the population. In order to further balance the optimal solution accuracy and optimization speed, the mutation factor is adjusted adaptively with the number of iterations. The numerical simulation results show that the proposed DePSO can realize the global path planning of the USV and achieved shorter path length than conventional PSO and DE. Compared with the existing methods, the method proposed in this paper is more suitable for the flight path planning of USV applying in water environment automatic inspection.

## Data availability statement

The original contributions presented in the study are included in the article/supplementary material, further inquiries can be directed to the corresponding author.

## Author contributions

YG proposed this contribution, verified, and concluded simulation results. ML gaves suggestions for simulation sets. SZ and SM gave suggestions for manuscript writing. All authors contributed to the article and approved the submitted version.

## Funding

This work was partially supported by the National Natural Science Foundation of China (NSFC) Under Grant Nos. U2106202 and 12075142, the Shandong Provincial Natural Science Foundation Under Grant No. ZR2020MA102, and the Science and Technology Planning Project of Zhejiang Under Grant No. 2020C03101.

## Conflict of interest

Author SM was employed by Zhejiang Jialan Ocean Electronics Co., Ltd. The remaining authors declare that the research was conducted in the absence of any commercial or financial relationships that could be construed as a potential conflict of interest.

## Publisher's note

All claims expressed in this article are solely those of the authors and do not necessarily represent those of their affiliated organizations, or those of the publisher, the editors and the reviewers. Any product that may be evaluated in this article, or claim that may be made by its manufacturer, is not guaranteed or endorsed by the publisher.
